# 3D Printed Polyurethane Scaffolds for the Repair of Bone Defects

**DOI:** 10.3389/fbioe.2020.557215

**Published:** 2020-10-23

**Authors:** Megan E. Cooke, Jose L. Ramirez-GarciaLuna, Karla Rangel-Berridi, Hyeree Park, Showan N. Nazhat, Michael H. Weber, Janet E. Henderson, Derek H. Rosenzweig

**Affiliations:** ^1^Biofabrication Laboratory, Research Institute of McGill University Health Centres, McGill University, Montreal, QC, Canada; ^2^Department of Surgery, McGill University, Montreal, QC, Canada; ^3^Bone Engineering Labs, Injury, Repair & Recovery Program, Research Institute McGill University Health Centres, McGill University, Montreal, QC, Canada; ^4^Department of Mining and Materials Engineering, McGill University, Montreal, QC, Canada

**Keywords:** mandibular defect, bone regeneration, polyurethane, 3D printing, layfomm, fused depositing modeling (FDM)

## Abstract

Critical-size bone defects are those that will not heal without intervention and can arise secondary to trauma, infection, and surgical resection of tumors. Treatment options are currently limited to filling the defect with autologous bone, of which there is not always an abundant supply, or ceramic pastes that only allow for limited osteo-inductive and -conductive capacity. In this study we investigate the repair of bone defects using a 3D printed LayFomm scaffold. LayFomm is a polymer blend of polyvinyl alcohol (PVA) and polyurethane (PU). It can be printed using the most common method of 3D printing, fused deposition modeling, before being washed in water-based solutions to remove the PVA. This leaves a more compliant, micro-porous PU elastomer. *In vitro* analysis of dental pulp stem cells seeded onto macro-porous scaffolds showed their ability to adhere, proliferate and form mineralized matrix on the scaffold in the presence of osteogenic media. Subcutaneous implantation of LayFomm in a rat model showed the formation of a vascularized fibrous capsule, but without a chronic inflammatory response. Implantation into a mandibular defect showed significantly increased mineralized tissue production when compared to a currently approved bone putty. While their mechanical properties are insufficient for use in load-bearing defects, these findings are promising for the use of polyurethane scaffolds in craniofacial bone regeneration.

## 1. Introduction

Critical sized bone defects are those that will not heal spontaneously, without intervention (Lichte et al., 2011). They can arise through trauma, poor fracture healing, and bone removal following severe infection or tumor resection. The current gold standard of treatment is to reconstruct the defect with autologous bone from a different region of the skeleton and to stabilize with ridged implants fixed to the bone. This has limitations including the amount of bone available for transfer without causing donor site morbidity, and increased risk of infection (Fairag et al., 2019). An alternative strategy for long term implantation, which has been used clinically, is to fill the defects with ceramic or resin-based pastes and to then secure them with implantable plates and screws (Williams, 2008). While pastes solidify to provide structural support for surrounding tissues, they are very dense materials and allow limited (if any) growth of new bone into the defect for repair (Lichte et al., 2011). The ideal scaffold is one which will provide initial structural support to the defect site and then be gradually degraded and replaced by newly formed bone.

The use of additive manufacturing (AM) is rapidly increasing in healthcare, particularly in the fields of dentistry and orthopedics (Liu et al., 2014; Dawood et al., 2015; Rosenzweig et al., 2015; Fairag et al., 2019). A key benefit of this approach is the potential for personalized implants. By reconstructing 3D scans of bony defects and reverse-engineering the damaged site, an implant can be produced of exact dimensions to repair the defect (Cox et al., 2016). There are numerous techniques and materials available such that metals, ceramics, and polymers can all be additively manufactured for orthopedic applications (Ahangar et al., 2019). This study investigates the use of fused deposition modeling (FDM) for bone reconstruction. This technique uses thermoplastics, usually supplied in filament form, that are heated directly before extrusion and then quickly cooled to solidify on the print bed (Zein et al., 2002). The hardware and materials for FDM are now readily available at relatively low cost and represent an economically viable technique to produce customized implants. A further advantage is the level of control over design parameters. For example, materials, macro-porosity and infill geometry can all be refined such that the scaffold can be tuned in terms of cell adhesion, cell infiltration, and stiffness, respectively (Nyberg et al., 2016).

LayFomm (PoroLay) is a polymer blend of polyvinyl alcohol (PVA) and polyurethane (PU). It is commercially available in 1.75 mm filament and can be printed at 215–225 ℃. Following printing, washing in water removes the water-soluble PVA, leaving a highly porous PU elastomer (Belka et al., 2017; Ahangar et al., 2018). This material has been used previously by our group to deliver therapeutic agents (Ahangar et al., 2018; Akoury et al., 2019). In this study we first investigated the use of LayFomm as a scaffolding material for the *in vitro* differentiation of dental pulp stem cells (DPSCs) and subsequent production of bone-like matrix. We then implanted the same material subcutaneously to determine the foreign body response to the material. Finally, scaffolds were implanted into mandibular defects in an *in vivo* rodent model to determine the potential for bony ingrowth and healing of the defect compared to a commercially available Norian cement used in craniofacial defects.

## 2. Materials and Methods

### 2.1. Preparation of Scaffolds

Scaffold blanks measuring 3 × 3 × 50 mm were designed in TinkerCAD (Autodesk, San Rafael, CA). For *in vitro* assessment and subcutaneous implantation they were designed with a central cavity of 750 μm with macropores in the walls measuring 750 μm in diameter every 5 mm. For mandibular implantation, there were no macropores. The models were exported as .stl files and sliced using Slicr3D. The blanks were printed with LayFomm60 (PoroLay Filaments, Germany) using a Duplicator i3 (Wanhao, China) using the following parameters: nozzle diameter 0.4 mm; nozzle temperature 215℃; print bed temperature 45℃; layer height 0.2 mm, print speed 10 mm.s^−^^1^. Following printing, individual scaffolds were cut from the blank to 5 mm lengths before being washed in dH_2_O four times to remove the water-soluble PVA. For cell seeding and implantation, scaffolds were disinfected by submersion in 70% ethanol for 4 h, followed by UV light exposure to each side for 20 min.

### 2.2. Scaffold Characterization

#### 2.2.1. LayFomm Filament Characterization

The composition of the LayFomm60 material is proprietary so to estimate the percentage of PVA and PU present, small pieces of filament were cut, weighed and then incubated at 37℃ in dH_2_O for up to 28 days (*n* = 4 for each timepoint). Following incubation, excess water was removed using a kimwipe before weighing to determine “wet” weight to account for swelling. They were then dried for 24 h at 37℃ before the “dry” weight was recorded. The change in weight was then calculated to determine when there was no further change.

#### 2.2.2. Mechanical Testing

3 mm long samples were loaded, perpendicular to the long fiber orientation, in unconfined compression at a rate of 0.045 mm.s^−^^1^ to 40% strain using a Mini Bionix 858 (MTS, Eden Prairie, MN). Compressive modulus was then calculated between 8 and 10% strain in the linear region of the curve.

#### 2.2.3. Scanning Electron Microscopy

Acellular samples were dehydrated through increasing concentrations of ethanol (70-80-90-95-100%) and then hexamethyldisilazane (HDMS, Sigma Aldrich, Oakville, ON) to dry overnight. Cell-seeded samples were fixed with 4% paraformaldehyde (PFA, Sigma Aldrich) for 1 h and then dehydrated through ethanol as above before being critical point dried using CO_2_ in a 030 CPD (Leica Microsystems, Richmond Hill, ON). Samples were coated with a 4 nm layer of platinum using a ACE600 high resolution sputter coater (Leica Microsystems) before being imaged using an FEI Quanta 450 ESEM (Thermo Fisher, Saint Laurent, QC).

### 2.3. *In vitro* Analysis of Scaffolds

#### 2.3.1. Seeding of Dental Pulp Stem Cells (DPSCs)

After disinfecting, as described previously, scaffolds were placed in sterile phosphate buffered saline (PBS, Sigma Aldrich) to maintain hydration. As previously reported (Fairag et al., 2019), 4 × 10^5^ DPSCs were suspended in 500 μL media and placed in a capped 3 mL syringe with two scaffolds. They were turned every 30 min for 2 h to ensure even coating of the scaffolds. After 2 h, scaffolds were moved into well plates, the excess media was centrifuged and cells that did not adhere were counted using a haemocytometer to determine seeding efficiency. Cell-seeded scaffolds were cultured in non-adherent multi-well plates with either control [high glucose DMEM (Sigma Aldrich) with pyruvate, glutamine and sodium bicarbonate with 1% Penicillin-Streptomycin (Gibco, Thermo Fisher), 10% heat-inactivated fetal bovine serum (Gibco) and 50 μg/ml ascorbic acid (Sigma Aldrich)] or osteogenic, OG (control media supplemented with 10 nM dexamethasone and 5 mM β-glycerol-2-phosphate) media. Cells were cultured on scaffolds for 21 days and media was changed twice weekly.

#### 2.3.2. Live/Dead Assay

After 21 days of culture, scaffolds were removed from media and washed with PBS. A 2 μM Calcein-AM, 4 μM Ethidium homodimer-1 (Invitrogen, Thermo Fisher) solution was prepared in PBS and applied to each scaffold for 15 min. Scaffolds were transferred to glass slides and imaged using an EVOS M5000 imaging system (ThermoFisher). Composite images were produced using ImageJ (NIH, Bethesda, MD).

#### 2.3.3. Crysectioning and Histology

Following live/dead imaging, samples were washed in PBS and then fixed in 4% PFA for 1 hr. They were then submerged in increasing concentrations of sucrose (10-20-30%) before being embedded in OCT (TissueTek, Sakura, Canada). When confident there were no bubbles in samples, they were snap frozen at -80℃. Gelatin-coated slides were prepared by dipping clean slides in a solution of 5% gelatin with 0.05% chromium potassium sulfate dodecadhydrate (Sigma Aldrich) before drying overnight. 10 μm sections were prepared using a CM1950 cryostat (Leica). Von Kossa, Alizarin Red (1% solution, Sigma Aldrich) and Safranin-O/Fast Green staining were then performed. Samples were mounted with Permount (Fisher Scientific) and imaged using an Axioskop 40 microscope with a high-resolution camera (Carl Zeiss, ON, Canada).

### 2.4. *In vivo* Implantation of Scaffolds

#### 2.4.1. Animal Maintenance

Live animal procedures were conducted in accordance with a protocol approved by the Facility Animal Care Committee of McGill University (AUP-7815) in keeping with the guidelines of the Canada Council on Animal Care, as previously described (Jabbour et al., 2014). Six- to eight-month-old male Sprague Dawley rats (Charles River laboratories, Senneville, QC, Canada) were caged individually and weighed weekly with unrestricted access to food and water.

#### 2.4.2. Subcutaneous Scaffold Implantation and Analysis

3 mm long × 2mm tall × 1 mm wide LayFomm scaffolds with 750 μm pores were 3D printed and disinfected as described previously (section 2.1). The scaffolds were inserted subcutaneously in the dorsum of four anesthetized rats between the shoulder blades through a 1 mm incision 5 mm away from the scaffold's final resting site. The incision was sutured using 4-0 PDS-II thread and the animals received 20 mg/kg/24 h carprofen for pain control for 3 days postoperatively. The scaffold was left undisturbed in place for 6 weeks after insertion.

Animals were euthanized by CO_2_ asphyxiation under anesthesia before the scaffolds, surrounding tissue and overlying skin were collected. They were fixed overnight with 4% PFA at 4℃, washed three times with cold PBS and embedded in paraffin for histological analysis. Five micron thick sections were prepared from the mid-sagittal point of the scaffold and stained with hematoxylin–eosin (H&E, Thermofisher - cat SH26-500D and cat 245-658, Waltham, MA, USA) to assess general morphology, and immunostained with alpha-smooth muscle actin (α-SMA, 1:300, abcam - cat 5694, ON, Canada), CD34 (1:300, abcam - cat ab23830), CD86 (1:300, abcam - cat ab238468) and Arginase-1 (1:300, Santa cruz - cat sc 271430, USA) to visualize fibrous tissues, vascular channels, M1 and M2 macrophages, respectively, using previously described methodology (Ramirez-Garcia-Luna et al., 2019). Images were captured with a Zeiss Axioskop 40 microscope (Carl Zeiss).

### 2.5. Mandibular Scaffold Implantation and Analyses

After 1-week of acclimatization, rats (*n* = 6) were anesthetized and the first molar was extracted on both sides. After a 4-week healing period, rats were randomly assigned to either LayFomm or Norian CRS putty (Kensey Nash, PA) implantation on each hemi-mandible. Norian CRS putty is a calcium phosphate bone cement clinically used for cranial repair and as such was deemed a clinically relevant comparator. The animals were again anesthetized to generate defects measuring 5 mm (sagittal) × 2 mm (frontal) × 3 mm (transverse) in the left and right mandibles using a 1 mm spherical burr (Stryker, Canada). All surgical procedures were performed with minimal trauma to preserve as much as possible the integrity of soft and hard tissues. Bone shards were washed away with gentle irrigation and either a 5 × 2 × 3 mm LayFomm scaffold (printed without macropores) or Norian CRS putty was inserted into the defects. A total volume of 100 μL per defect were used to fill the void by press fitting the putty into it. The residual cement was gently wiped with gauze, ensuring the void remained full. LayFomm scaffolds were press-fitted into the defects. In both cases, the gums were sutured to maintain the materials in place. All rats were given soft food (DietGel Recovery, ClearH2O, ME) *ad libitum* and 20 mg/kg/24 h carprofen for pain control for 3 days postoperatively. Rats were then switched back to regular chow and maintained for 6 weeks. Immediately after animal euthanasia, by CO_2_ asphyxiation under anesthesia, the region of interest of the mandibles was carefully extracted and excess soft tissue removed before fixation for 24 h in 4% PFA at 4℃. The 6-week post-implantation time point was selected because it lays in the coupled-remodeling stage of bone healing. Moreover, from our previous experience, defects that at this point have not been filled with bone will most likely develop fibrous non-unions, thereby being a good time point to assess long-term outcomes (Ramirez-Garcialuna et al., 2017).

#### 2.5.1. Micro-CT Analyses

Rat mandibles with inserted scaffolds were carefully dissected free of soft tissue, fixed for 24 h in 4% paraformaldehyde at 4℃ and then rinsed thoroughly with sterile PBS prior to micro-computed tomography (μCT) analysis. A skyscan 1172 (Bruker, Milton, 139 ON) was used with 9 μm/pixel resolution, using a 1.0 mm aluminum filter at a voltage of 59 kV and a 140 current of 167 μA. 2D projections were reconstructed into slices using NRecon (Bruker) and analyzed using CTAn v.1.16.4.1 (Bruker). 3D reconstructions were visualized using CTVol (Bruker). Quantitative data for bone regeneration was recorded in a region of interest (ROI) measuring 5 mm long × 3 mm wide × 2 mm deep, in the middle of the bone window defect, encompassing the defect and scaffold. Quantitative data for mineralized tissue includes bone quantity (BV/TV), trabecular number (Tb.N), trabecular thickness (Tb.Th), separation of trabeculae (Tb.Sp), connective density (Conn.Dn), total porosity (Po.Tot), and structure model index (SMI) (Drager et al., 2017).

#### 2.5.2. Histological Analyses

Following micro CT, mandibles were decalcified in 10% EDTA for 21 days before embedding in paraffin as previously described (Ramirez-Garcialuna et al., 2017). Serial 5 μm sections were cut in the sagittal plane in the implant region. Sections were probed for alkaline phosphatase (ALP) activity in osteogenic cells, tartrate resistant acid phosphatase (TRAP) activity in catabolic cells (Abcam, Cambridge UK). Samples were imaged using an Axioskop 40 microscope with a high-resolution camera (Zeiss).

### 2.6. Statistical Analysis

Non-linear curves were fit to the dry and change in weight of filament samples, while a spline fit (7 knots) was used to fit a line to the wet weights. Error bars or line fills indicate standard deviation from the mean. Paired t-tests were performed between compressive moduli and microCT data. Values of *P* < 0.05 were deemed statistically significant. All statistical analyses were performed using GraphPad Prism v8.

## 3. Results

### 3.1. Characterization of LayFomm Material and Acellular Scaffolds

**Figure 1:**
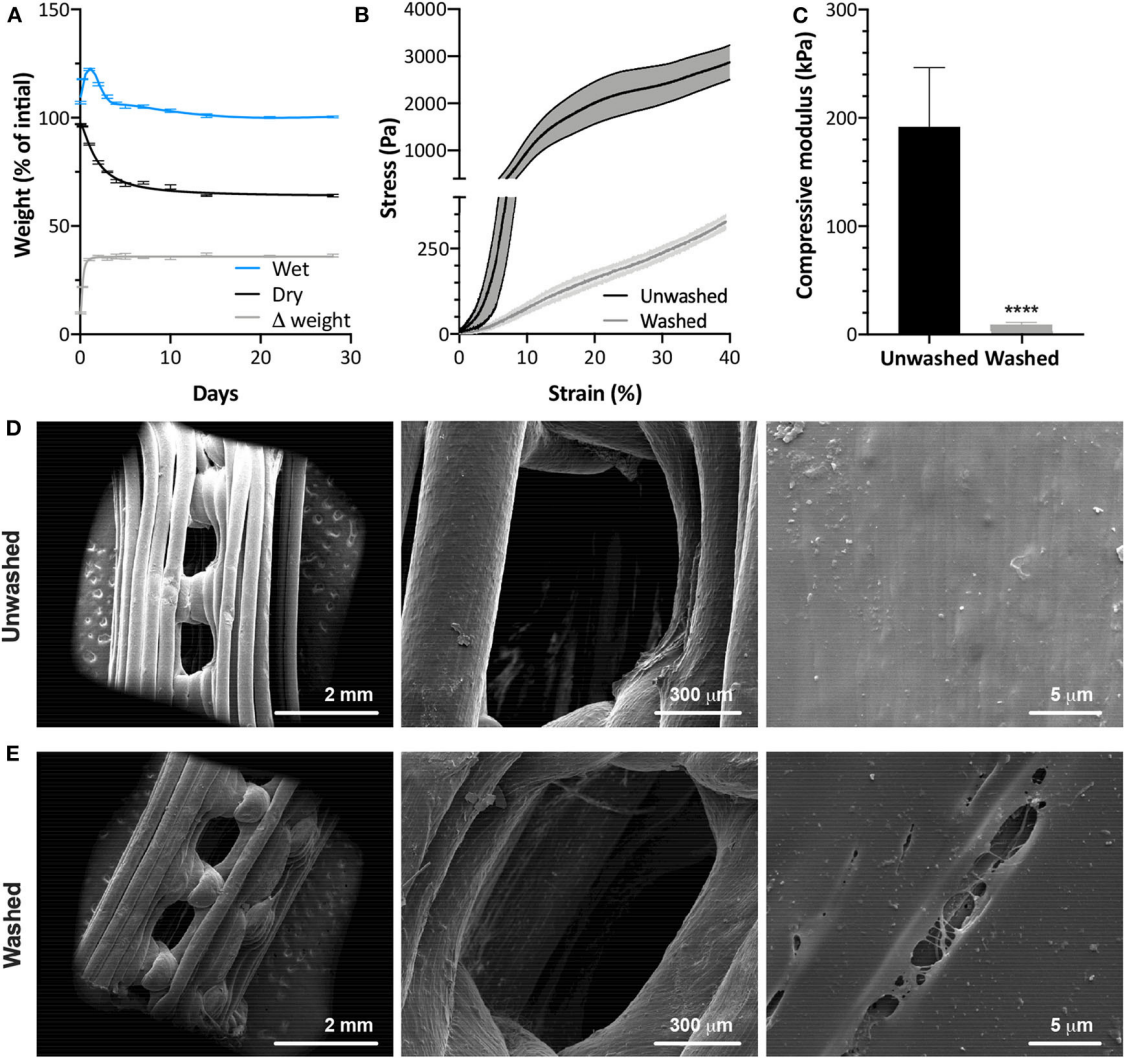
**(A)** Temporal change in scaffold weight with removal of PVA by washing in dH_2_O (*N* = 4). Mechanical compression data of washed and unwashed scaffolds showing **(B)** deformation behavior and **(C)** compressive modulus between 8 and 10% strain (*N* = 5). SEM images of **(D)** washed and **(E)** unwashed LayFomm scaffolds, showing the appearance of micropores (arrowheads) following removal of PVA by washing in dH_2_O. Error bars **(A,C)** and fill between lines **(B)** indicates standard deviation. For **(A)**, lines are as follows: wet—smoothing spline, 7 knots; dry and Δ weight—non-linear fit, *R*^2^ = 0.9907 and 0.9892, respectively. ****P < 0.0001.

The percentages of PVA and PU were estimated by incubating small pieces of LayFomm60 filament in water at 37℃ for up to 28 days. As shown in Figure 1A, after the initial swelling phase, there is a consistent difference (Δ) in dry and wet weights of 36% between 4 and 28 days. The initial removal of water-soluble PVA happens quickly, with the dry mass decreasing by 4% after just 1h and continuing to decrease, down to a plateau of 64% of the original mass between 14 and 28 days (mean d14 = 64.01, d21 = 64.08, d28 = 64.01%). This plateau suggests that all PVA has been removed and there was no degradation of PU between 14 and 28 days.

Compression testing shown in Figures 1B,C reveals that removal of the PVA causes a significant reduction in the mechanical strength of the scaffolds. The stress-strain curves in Figure 1B, show that before washing, there is a steep gradient to the curve in the elastic region and evidence of a yield point prior to the region of plastic deformation. Following washing, however, there is clear elastomeric behavior. There is a small decrease in gradient of the curve around 15% strain, likely when the pores of the scaffold have been completely compressed, but no clear yield point. The gradient then increases again without plateauing up to 40% strain. Figure 1C shows a significant reduction in compressive modulus following washing. Removal of the PVA had no effect on the macrostructure of the LayFomm scaffolds. It did, however, result in micropores ranging from approximately 200 nm to 20 μm visible on the surface (Figures 1D,E).

### 3.2. *In vitro* Analyses

**Figure 2:**
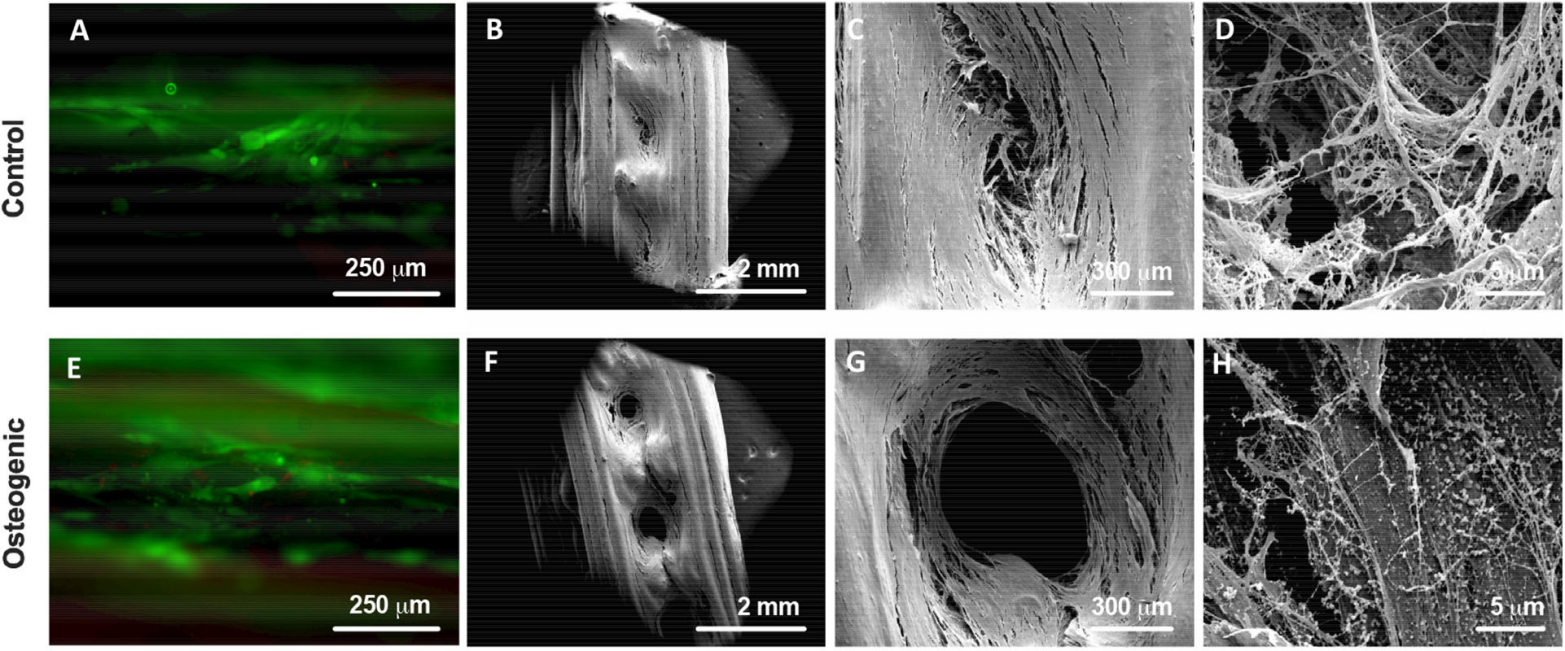
Live/Dead staining **(A,E)** showing good viability of DPSCs on the scaffolds. SEM images of DPSC-seeded scaffolds after 21 days of culture in either control **(A–D)** or osteogenic **(E–H)** media showing cells and matrix filling the macropores of the scaffolds.

Initial seeding had a 68% adhesion success, resulting in approximately 1.36 × 10^5^ cells seeded onto each scaffold (data not shown). After 21 days of culture, DPSCs in both control and osteogenic media proliferated, showed very good viability (Figures 2A,E) and produced matrix that filled the pores of scaffold (Figures 2B,C,F,G). DPSCs cultured in osteogenic media, showed some evidence of mineralized matrix formation, with small crystals visible under SEM (Figure 2H) compared to control media (Figure 2D).

**Figure 3:**
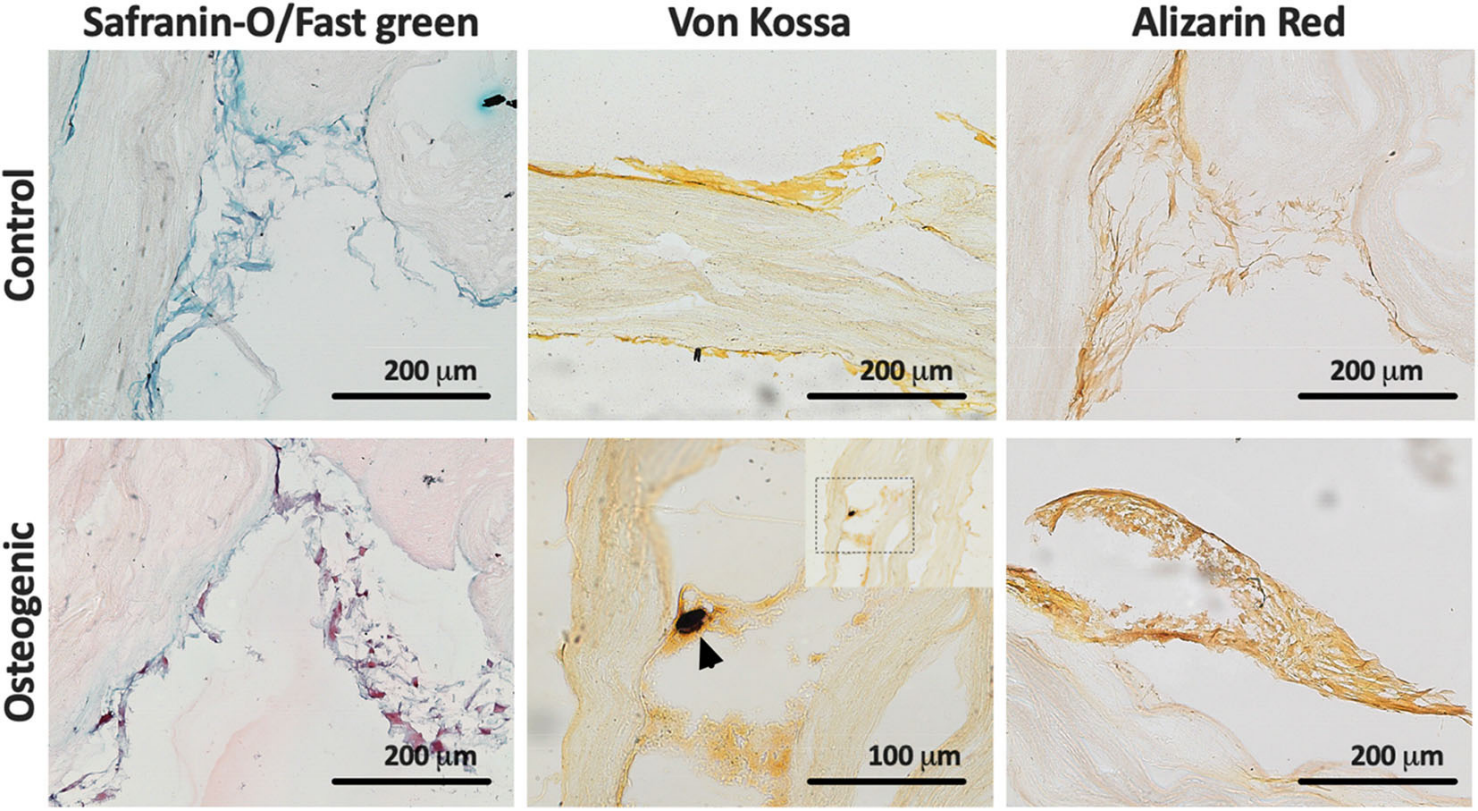
Histology of DPSCs cultured on LayFomm scaffolds in either control or osteogenic media for 21 days. Safranin-O/Fast green staining indicates formation of collagenous matrix in both conditions; Von Kossa staining for phosphate, arrows indicate phosphate-rich nodules formed in osteogenic media; Alizarin Red staining for calcium shows increased straining in osteogenic media.

Histological evaluation of LayFomm scaffolds cultured with DPSCs for 21 days is presented in Figure 3. In all staining, the produced matrix is clearly visible. Safranin O/Fast green staining shows production of collagen-rich matrix in both conditions as would be expected. Von Kossa staining shows some evidence of phopsphate-rich nodules as indicated by the arrowhead in Figure 3 when DPSCs were cultured in osteogenic media. Alizarin Red S staining is slightly increased with osteogenic media, indicating increased production of calcium-rich matrix.

### 3.3. *In vivo* Subcutaneous Implantation

**Figure 4:**
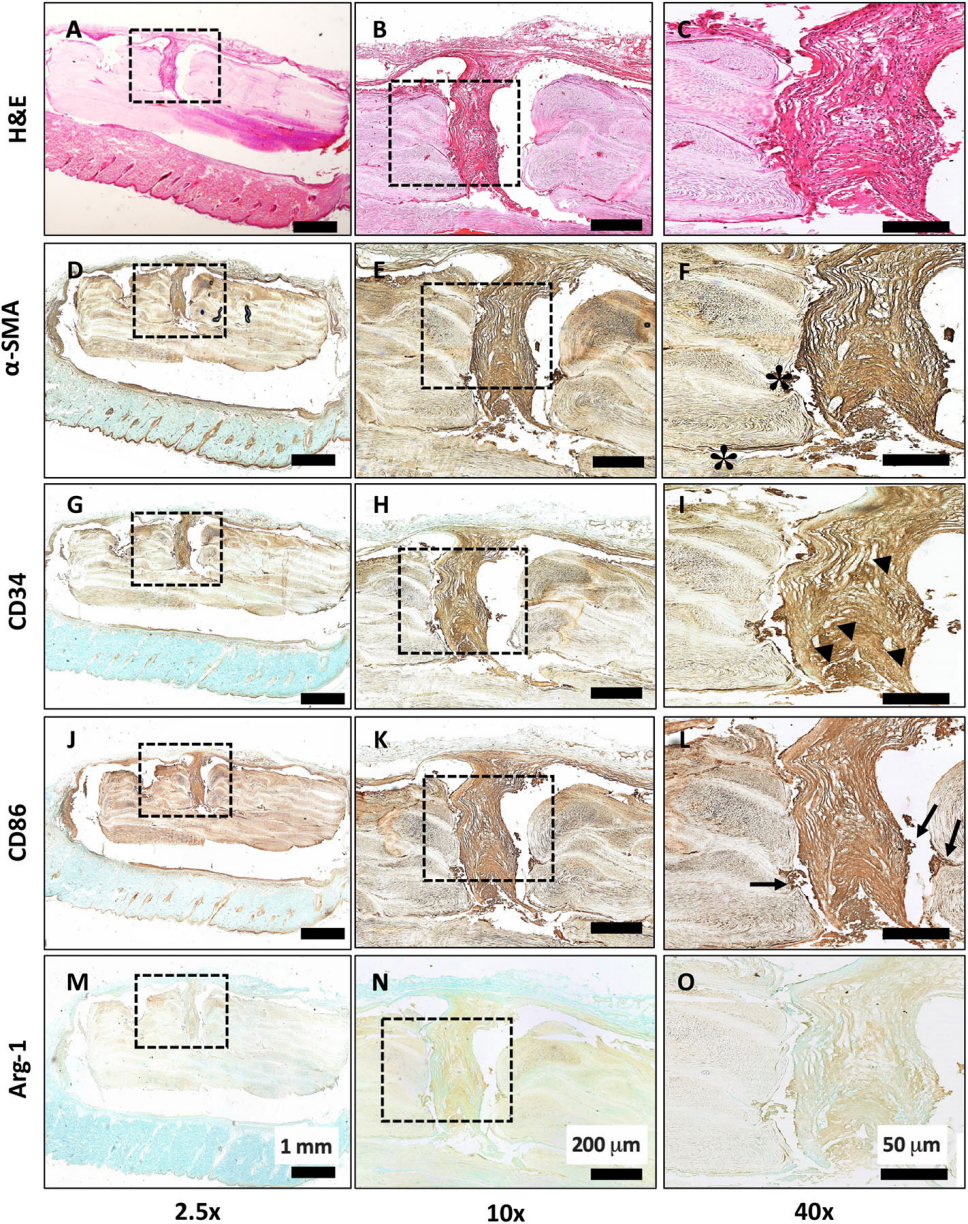
Histological evaluation of subcutaneous implantation of LayFomm scaffold. **(A–C)** H&E staining of overall tissue morphology; **(D–F)** α-smooth muscle actin shows formation of fibrous tissue around and directly next to (*) the implanted scaffold; **(G–I)** CD34 shows positive staining for haematopoietic stem cells, indicating vascularization (arrowheads); **(J–L)** CD86 staining shows few clusters of M1 macrophages at the scaffold-tissue interface (arrows); **(M–O)** Arg-1 staining for M2 macrophages is negative.

Scaffolds were first implanted subcutaneously for 6 weeks to determine any local inflammatory response. In Figures 4A–C, H & E staining shows the overall morphology and presence of fibrous tissue growing into the pores of the scaffold. There were no necrotic regions observed. Strong staining of α-smooth muscle actin (α-SMA) in Figures 4D–F confirms the activation of a fibrotic response to the LayFomm scaffold. Figures 4G–I show positive staining for CD-34, showing the presence of haematopoietic stem cells that indicates blood vessel formation. CD86 staining in Figures 4J–L shows clusters of M1 macrophages at the implant-tissue interface, showing that the material is not biologically inert and there is a mild inflammatory response. Finally, in Figures 4M–O, negative arginase-1 staining confirms the absence of M2 macrophages in the fibrous tissue formed. This shows that the scaffold did not promote a chronic inflammatory response.

### 3.4. *In vivo* Mandibular Implantation and Bone Ingrowth

**Figure 5:**
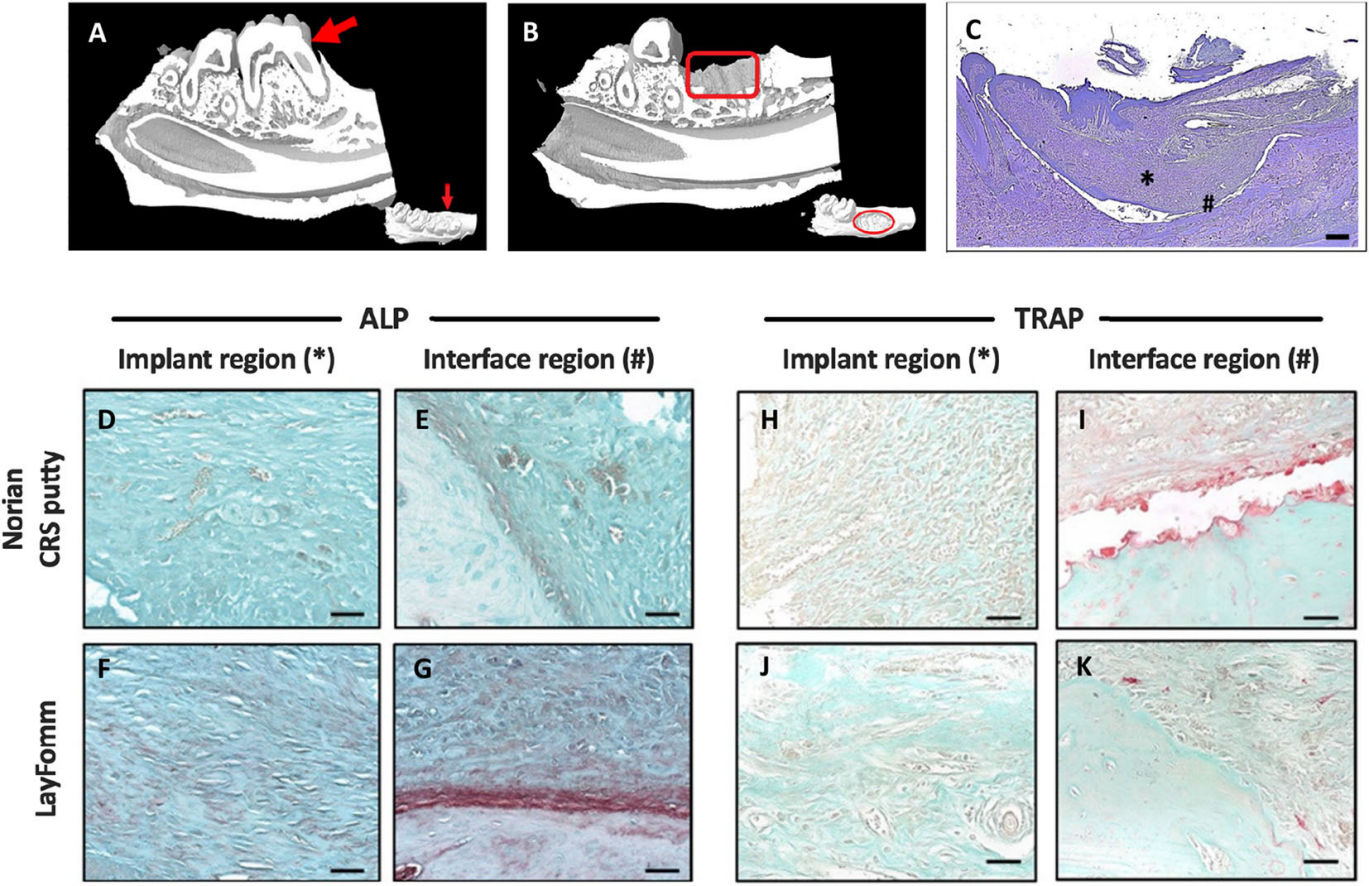
MicroCT reconstruction of rat mandible pre **(A)** and post **(B)** implantation of LayFomm scaffold; LayFomm is radio-translucent so not visible. **(C)** Toluidine Blue staining of the scaffold following implantation. *Implant region and ^#^bone interface region in **(D–K)**. ALP **(D–G)** and TRAP **(H–K)** staining of repaired tissue and the bone interface following 6 weeks of either Norian putty or LayFomm implantation. Scale bar = 50 μm.

Following molar extraction and a 4 week recovery period a 5 mm defect was drilled in the mandible. Figure 5 shows the pre- (Figure 5A) and post-operative (Figure 5B) *in vivo* MicroCT of the molar extraction and scaffold implantation. LayFomm is polymeric and thus radio-translucent so not visible by CT. Toluidine Blue staining in Figure 5C shows the scaffold in place, regions of repaired tissue in the scaffold is marked by an asterisk (*) and the bony interface is shown by a hash (#).

Histological staining of the recovered tissues was performed to investigate bone formation in the implanted scaffolds. Alkaline phosphatase staining was positive in both the Norian CRS putty and LayFomm groups (Figures 5D–G). Stronger staining in the LayFomm group indicates increased levels of osteogenesis (Figures 5F,G). TRAP staining for osteoclast activity was much higher at the bone interface in the Norian putty (Figure 5I), such that the putty appears to have been resorbed away from the native bone. In the LayFomm group however, the interface between the scaffold and native bone is constant (Figures 5J,K). This indicates that there is a less of an inflammatory response with the implantation of LayFomm compared to the Norian putty.

**Figure 6:**
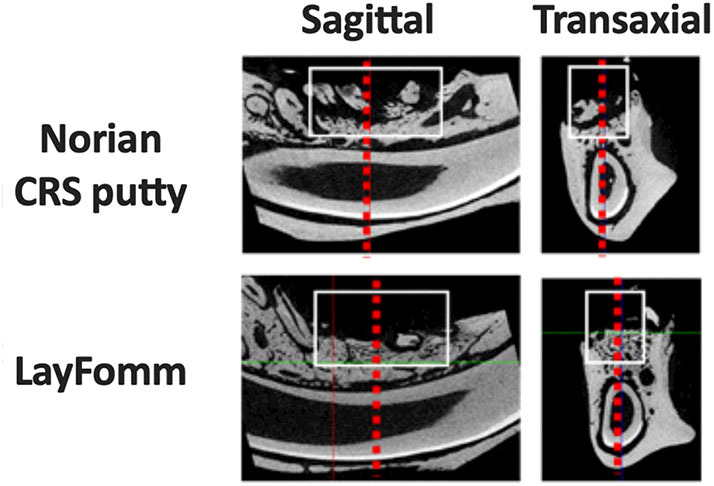
MicroCT reconstructions of implanted scaffolds compared to Norian CRS putty. The white box marks the analyzed ROI that corresponds to data in Table 1. Dashed red lines represent the corresponding transaxial and sagittal views.

Finally, analysis of the microCT reconstructions was performed (Figure 6 and Table 1). Quantitative μCT analysis showed a significantly increased amount of mineralized tissue (BV/TV) in the mandibles implanted with LayFomm scaffolds compared with those implanted with Norian CRS Putty. This increase in bone mass was reflected by significantly higher trabecular thickness (Tb.Th) that exhibited less separation (Tb.Sp), and a significantly different geometry (SMI).

**Table 1:** Bone volume (BV), Bone volume/Tissue volume (BV/TV), numbers of trabeculae (Tb.N), trabecular thickness (Tb.Th), trabecular separation (Tb.Sp), Total porosity (Po.Tot), Connective Density (Conn.Dn), Structure Model Index (SMI).

**Parameter**	**Norian CRS Putty (*n* = 6)**	**LayFomm (*n* = 6)**	***P*-value**
BV/TV (%)	14.33 ± 7.94	30.26 ± 9.46	0.02
Tb.Th (μm)	140.5 ± 14.3	201.7 ± 33.4	<0.001
Tb.Sp (μm)	1456.0 ± 226.9	685.5 ± 113.3	<0.001
Tb.N (no./mm)	1.66 ± 0.65	1.58 ± 0.58	0.69
Po(Tot) (%)	76.92 ± 8.56	69.74 ± 9.46	0.16
Conn.Dn (1/μm)	0.09 ± 0.06	0.11 ± 0.04	0.55
SMI	−3.92 ± 1.18	−0.30 ± 1.68	<0.001

*Data was analyzed using paired t-tests (n = 6 rats)*.

## 4. Discussion

Critical-sized bone defects are a challenging scenario for clinicians and patients. A scaffolding material that allows for bony regrowth while providing structural support to the bone has the potential to help millions of patients around the world annually. The rapidly increasing availability of additive manufacturing hardware is likely to have a huge impact on the medical field. FDM printers are already becoming available in hospitals for surgical planning and education but the use of AM in surgical practice remains limited. This is due in part to the lack of highly qualified personnel to perform the computational tasks that convert patient scans to 3D models for printing. As technological developments enable the automation of this critical step, implantable devices will be designed, manufactured and sterilized within the confines of surgical units.

A large range of materials are currently available for additive manufacturing, that include metals, ceramics and polymers. Metallic implants have commonly been used in orthopedic applications due to their inherent stiffness; they have traditionally been used for long-term structural applications but recent studies are seeking to increase their biological applications (Cox et al., 2016, 2017; Burton et al., 2019). A number of additively manufactured titanium implants have now been FDA approved, such as the FastForward device for correction of hallux valgus deformities (Smith et al., 2016). Polymers are a group of materials that have many different characteristics including cell adhesion, degradation rate and mechanism. Multiple polymers can be blended or co-polymerized to alter their properties. The material used in this study, LayFomm, is a blend of PU and PVA. The PVA acts as a stiffener, such that the blend can be extruded into filament and then printed using FDM. The water-soluble PVA can then be washed away leaving just the highly swollen functional elastomer, PU.

The PU used in this study allowed for rapid cell attachment, as seen by a 68% seeding efficiency after just 3 h. This may be attributed to the hydrophilicity of the material and also its microporosity (Marzec et al., 2017). Microporosity gives a larger surface area and has been shown to increase protein adhesion, cell adhesion and proliferation as well as playing a critical role in osteogenesis in bone scaffolds (Muschler et al., 2004; Liu et al., 2013; Zhang et al., 2018). This microporosity in LayFomm has previously been exploited as a means to enable uptake and also deliver small molecules. We have shown the ability for LayFomm to uptake and then release chemotherapeutics over a period of 14 days (Ahangar et al., 2018), while other groups have used LayFomm in separation science. Konieczna et al. (2018) used LayFomm to entrap small molecules such as steroids from human plasma prior to analysis in mass spectroscopy. As such, it would be possible to load osteoinductive factors such as bone morphogenic proteins (BMPs) into these scaffolds prior to implantation. This concept has been shown to promote bone formation *in vivo*; Bouyer et al. showed complete bridging of critical-sized defects in a rat models with BMP-2 coated PLGA and PCL scaffolds (Sawyer et al., 2009; Bouyer et al., 2016).

Dental pulp stem-cells were used in this study for *in vitro* analysis due to their fast proliferation rate and ability to undergo osteogenic differentiation in the presence of the appropriate factors (Gronthos et al., 2000; Zhang et al., 2006). In this study they were able to proliferate, differentiate and produce matrix, filling the 750 μm macropores in the scaffold; pores of this size-range have been shown to favor cell migration *in vitro* (Karageorgiou and Kaplan, 2005; Fairag et al., 2019). Similarly, after subcutaneous implantation *in vivo*, fibrous tissue was shown growing into the macropores of the scaffold. A mild inflammatory response is required for the integration of a foreign material into a biological system. A fibrotic response was seen here by the positive staining of α-smooth muscle actin. A mild inflammatory response was observed by the positive staining of M1 macrophage marker CD-86, while there was no chronic inflammation as evidenced by the absence of M2 macrophages. Macrophages play a pivotal role in the foreign body response (Klopfleisch, 2016), they have been shown to be responsible for the recruitment of vascular cells, enabling angiogenesis (Spiller et al., 2014). Formation of a vascular network is critical in bone regeneration, as seen in the healthy fracture healing response (Marsell and Einhorn, 2011). Subcutaneous implantation showed evidence of vascularization in the fibrous tissue formed around the implant (by CD34 staining). In the mandibular defect model, there was limited evidence of CD34 staining; a possible reason for the lack of vascularization is the lack of porosity in the scaffolds used (these scaffolds were printed with 100% infill density). It has been shown previously that without macropores, there is a lack of interconnectivity for growth of a vascular network (Hutmacher, 2000; Liu et al., 2013).

Formation of calcified matrix on the PU scaffold was shown both *in vitro* using SEM and also histologically, by the presence of phosphate-rich nodules in Von Kossa staining. *In vitro*, osteogenic media was required to promote this response despite DPSCs having many bone-like biochemical markers (Gronthos et al., 2000). Mineralized matrix formation *in vivo* was shown by micro-CT; the significant increase in BV/TV compared to the Norian putty is promising for its use as a 3D printed scaffold for bone regeneration. Polyurethanes have previously been shown to promote calcification *in vivo* and the mechanism has been proposed as by the interaction of PU with calcium and phosphate in the blood and other fluids (Marzec et al., 2017). PU is hydrophilic and has polar groups resulting in high affinity for CaP binding (Jie and Yubao, 2004). The composition of PU in the LayFomm filament was estimated by a 28-day washing study, with a plateau in dry mass at 64% of the initial weight after 14 days. An important limitation of this method to determine the amount of PU/PVA in the LayFomm filament is that it does not differentiate between degradation of PU and solubilization of PVA in this time frame. The plateau in dry mass between days 14 and 28, however, suggests that there is no degradation in this time. The formation of bone-like tissue is encouraging, however, a key limitation of LayFomm as a bone scaffold is its low mechanical stiffness. This is an important reason for the use of a mandibular defect rodent model in this study, rather than a load-bearing critical defect model.

This is the first study to characterize LayFomm as a potential material for bone regeneration both *in vitro* and *in vivo*. The successful formation of mineralized matrix is promising for this as a bone repair strategy. As an elastomer, the mechanical stiffness of this scaffold is not high enough to be utilized as a scaffold in a load bearing application but may be useful in craniofacial defect repair. An interesting avenue for further investigation is in delivery of therapeutics within the micropores of the material to further enhance its capacity for bone formation.

## Data Availability Statement

The raw data supporting the conclusions of this article will be made available by the authors, without undue reservation.

## Ethics Statement

The animal study was reviewed and approved by Animal Care Committee of McGill University (AUP-7815).

## Author Contributions

DR and JH: conception of study. MC: manuscript preparation. MC, KR-B, JR-G, and HP: experimental work and data analysis. DR, JH, and SN: supervision. All authors contributed to the article and approved the submitted version.

## Conflict of Interest

The authors declare that the research was conducted in the absence of any commercial or financial relationships that could be construed as a potential conflict of interest.
